# A Hybrid Kinematic-Acoustic System for Automated Activity Detection of Construction Equipment

**DOI:** 10.3390/s19194286

**Published:** 2019-10-03

**Authors:** Behnam Sherafat, Abbas Rashidi, Yong-Cheol Lee, Changbum R. Ahn

**Affiliations:** 1 Department of Civil and Environmental Engineering, University of Utah, Salt Lake City, UT 84112, USA; abbas.rashidi@utah.edu; 2 Department of Construction Management, Louisiana State University, Baton Rouge, LA 70803, USA; yclee@lsu.edu; 3 Department of Construction Science, Texas A&M University, College Station, TX 77843, USA; ryanahn@tamu.edu

**Keywords:** construction equipment, audio and kinematic signals, sensor fusion, dimension reduction, activity detection, support vector machines

## Abstract

Automatically recognizing and tracking construction equipment activities is the first step towards performance monitoring of a job site. Recognizing equipment activities helps construction managers to detect the equipment downtime/idle time in a real-time framework, estimate the productivity rate of each equipment based on its progress, and efficiently evaluate the cycle time of each activity. Thus, it leads to project cost reduction and time schedule improvement. Previous studies on this topic have been based on single sources of data (e.g., kinematic, audio, video signals) for automated activity-detection purposes. However, relying on only one source of data is not appropriate, as the selected data source may not be applicable under certain conditions and fails to provide accurate results. To tackle this issue, the authors propose a hybrid system for recognizing multiple activities of construction equipment. The system integrates two major sources of data—audio and kinematic—through implementing a robust data fusion procedure. The presented system includes recording audio and kinematic signals, preprocessing data, extracting several features, as well as dimension reduction, feature fusion, equipment activity classification using Support Vector Machines (SVM), and smoothing labels. The proposed system was implemented in several case studies (i.e., ten different types and equipment models operating at various construction job sites) and the results indicate that a hybrid system is capable of providing up to 20% more accurate results, compared to cases using individual sources of data.

## 1. Introduction

In the construction industry, three important resources at job sites are crews, materials, and equipment. For the majority of construction projects, a major expense item is the budget allocated to the acquisition, rental, and maintenance of heavy equipment [1]. The costs depend on several factors, including the idle and working hours, as well as productivity and efficiency rates. When it comes to working hours and productivity rates, two types of problems can occur during equipment working shifts. One is idle time, or the lack of operation while the equipment engine is working. This situation can result from poor planning (e.g., lack of a sufficient number of dump trucks to be loaded by an excavator), human factors, etc. The second type of problem is downtime when equipment is inactive, due to mechanical/electrical problems and the need to be repaired.

Carefully analyzing and monitoring heavy equipment productivity rates and monitoring downtimes, idle times, and productive times are well-known significant factors in the success of construction projects. Traditional methods for productivity analysis and construction equipment and machinery performance monitoring are through direct observations, as well as conducting interviews and surveys. These methods are labor-intensive and prone to error, making them impractical for larger job sites and more complex environments. As a result, there is an increasing demand for more efficient and systematic solutions for productivity analysis of heavy equipment under various conditions [2]. 

Construction equipment productivity rates are directly associated with various activities performed by the machine during routine operations. Recognizing these activities is the first step toward analyzing efficiency rates [3,4]. A systematic activity recognition system could help project managers (1) evaluate machines idle versus working times, (2) estimate cycle times of construction operations, and (3) analyze productivity rates [5,6].

Recent advancements in technology motivated researchers to develop automated systems to decrease human interference [7,8,9]. In parallel with recent advancements in developing computing methods and Information Technology (IT) tools, researchers have devised several techniques for automated equipment activity detection in construction job sites. These techniques could be divided into three major categories. The first category uses vision-based technologies, such as two-dimensional (2D)/image and three-dimensional (3D) range cameras, and utilizes computer vision algorithms to analyze images and videos [10,11,12]. The second category employs kinematic sensors, such as accelerometers and gyroscopes, to record the equipment’s kinematic signals and detect its activities [13,14]. The third method is based on processing audio signals [1,15,16], using single microphones or microphone arrays to record sound patterns generated by machines while performing various operations. 

The studies mentioned above use one of the three major sources of data (i.e., visual, kinematic, or acoustic data) to detect equipment activities. Each type of data, as well as the associated processing algorithms, have certain limitations and drawbacks. For computer vision-based methods, factors such as digital cameras’ limited field of view, illuminations, and occlusions, could negatively impact the widespread use of this category at complex construction job sites. For the case of audio-based methods, certain types of construction machinery do not generate distinct sound patterns during the course of operation (e.g., cranes, soil compactors, etc.), so relying on audio as the only source of data might not always be a feasible solution. Finally, for the kinematic-based methods, it could be challenging to directly attach kinematic sensors to the body of the machine (e.g., jackhammers, concrete pumps, concrete truck mixers, etc.).

To address the distinct weaknesses of these existing methods, the authors have investigated the feasibility of integrating two major sources of data, kinematic and acoustic, and developing a hybrid system for activity detection of construction equipment. The proposed hybrid system is based on a combined feature-based approach to increase the final results’ accuracy. Several types of features are extracted from both data sources and merged to generate more accurate results. The presented system is capable of detecting a wide range of activities taking place at construction job sites, such as moving forward/backward, arm raising/lowering, rotating, loading and excavating, etc. Compared to using individual sources of data, a hybrid system can cover a broader range of equipment types and job site conditions, eventually generating more accurate results. 

This paper is organized as follows: Section 2 presents a broad overview of activity detection methods in different areas. A more in-depth literature review on various methods for construction equipment activity detection will also appear in this section. In Section 3, we provide technical details about the proposed hybrid system. Details of experimental setups and obtained results will be presented in Section 4. Finally, Section 5 includes the conclusions and lessons learned from this study.

## 2. Literature Review

As indicated earlier, two major methods for equipment activity detection are based on kinematic or audio signals. In the following sections, various studies in the general field of audio recognition and activity detection are examined. Then, we discuss recent studies on equipment activity detection using audio data. In the following section, we investigate general activity detection studies using kinematic signals. These studies have utilized various types of features, machine learning models, and evaluation processes. We also review more related studies in the domain of construction, especially equipment activity detection. Finally, in the last section, we provide a comprehensive comparison between these two methods and discuss the role of data fusion.

### 2.1. Audio Recognition and Activity Detection Using Audio Signals

Audio carries useful information about our surroundings. This particular source of data could be used to identify events that occur in our environment for Enhanced Living Environments (ELE) and Ambient Assisted Living (AAL) [17]. With the advent of new methods and tools, sound recognition and classification have attracted a significant amount of attention from researchers and practitioners. Audio recognition and classification consist of extracting different features from a sample audio file and feeding these features into a machine-learning algorithm, to detect classes of the present sounds. This topic has been studied for ambient sound classification [18,19,20], noise signal classification [21,22], speech/music classification [23], music genre classification [24], human accent or language classification, speaker recognition [25,26], and indoor localization [27]. Hinton et al. [28] and McLoughlin et al. [29] used Deep Neural Network (DNN) to develop an automated speech recognition system and a robust sound event classification, respectively. Similarly, Graves et al. [30] utilized Deep Recurrent Neural Networks (DRNN) for speech recognition and sound event classification. Hwang and Lee [31] developed a crowdsourcing framework to obtain real-life data from the environment to detect different types of activities occurring in the surroundings. The k-nearest neighbors’ method has been used in their system, however, their reported accuracies are not sufficiently high.

Few studies have been conducted on equipment activity detection using audio signals. In References [1,2,15,16,32,33], audio signals have been used to detect construction equipment activities. In those studies, major activities were defined as those directly contributing to the productivity of the project (e.g., loading, pushing soil, excavating, etc.). Also, those studies only utilize the magnitude of Short Time Fourier Transform (STFT) of audio signals as the input feature, and the proposed systems are only applicable for equipment that generates distinctive sound patterns. This situation is additionally problematic since the presence of any sound barriers might affect the output’s accuracy. In a complementary effort to fix some of those issues, Cheng et al. [2] studied necessary hardware and software settings for an audio-based equipment activity detection system to obtain more rigorous results. They compared different types of microphones and locations to achieve optimal solutions for their system. Cheng et al. [34] proposed an approach to label weakly labeled data, based on the clustering behavior of audio features in data frames.

### 2.2. Activity Detection Using Kinematic Signals

Unlike audio, kinematic data has been commonly utilized for activity recognition purposes in different areas. Kinematic sensors are cost-effective and easy-to-use devices which can be conveniently used for activity detection purposes, especially detecting human beings’ various activities. Several studies have been conducted in the last two decades regarding human activity detection [35,36,37,38,39,40,41,42]. Since human activity detection is not the focus of this paper, only a few recent papers on this subject are discussed in this section. In one of the most relevant studies, Ignatov and Strijov [43] utilized smartphone accelerometer data and the fundamental periods’ extraction method to detect six types of human activities. Another application of human activity detection is gait analysis. Anwary et al. [44] created an app for this purpose after investigating different locations for optimal sensor placement. They used both accelerometer and gyroscope data and extracted ten different features. Their results indicated that the orientation and location of sensors could significantly impact the results. Lee and Kwan [45] integrated Global Positioning System (GPS) and accelerometer data to detect the location and type of human activities. They extracted 59 features and applied two different approaches, i.e., 10-fold cross-validation and random forest.

In recent years, several studies have been conducted to detect equipment activities and measure the performance of construction operations using kinematic sensors. Ahn et al. [46] proposed a monitoring system to determine the equipment’s operational efficiency and environmental performance based on kinematic signals. By observing signal energy, idle periods of equipment are determined. This research only uses signal energy as a feature and does not use any machine learning models or signal processing techniques. Moreover, it was tested on one piece of equipment (e.g., excavator), and cannot be generalized to all other types of equipment in real-world construction job sites. Akhavian and Behzadan [47] developed an automated model to track equipment and determine its different motions. The model detects relative movements of different parts of the equipment using magnetic field and tilt sensing and creates a real-time 3D animation. The output of their system can be used for long-term planning and does not detect various activities handled by the equipment for performance monitoring. Later, in References [48,49], Akhavian and Behzadan fused different data types—such as weight, position, and orientation—to detect varying equipment activities’ duration and also determine properties of the queuing system to be used in a simulation model. Through a separate research study [46], the authors utilized accelerometer, gyroscope, and GPS data for detecting heavy construction equipment activities. They have not reported any pre-processing methods on sensor data and used a few time-domain and frequency-domain features, which led to moderate accuracies that may require further improvements. In a similar effort, Ahn et al. [14] developed a method based on accelerometer data to detect three different classes of activities: engine-off, idling, and working modes. Their proposed method was preliminarily tested on excavators. In one of the most recent studies, Kim et al. [50] implemented Inertial Measurement Units (IMUs) to detect an excavator’s various activities. They used the concept of Dynamic Time Wrapping (DTW) to increase the accuracy rates to detect mixed activities. This research mainly focused on cabin rotation to determine process cycle time.

### 2.3. Audio and Kinematic Data Comparison

As indicated earlier, the existing studies in the literature often used either audio or kinematic data as the primary source of information for automated activity detection of construction equipment. There are some limitations and restrictions in using each of these data sources. Due to sound barriers and long distances between the microphone and the sound’s source, audio might not be a useful source in large job sites. Moreover, some newer equipment models perform quietly and might not generate distinctive sound patterns necessary for further processing. A similar issue may exist for kinematic signals where new equipment might not generate strong vibration signals. Also, some types of equipment do not offer proper space to place kinematic sensors or mobile phones (e.g., small hand drills). Another issue within the existing methods is that in most current studies, only a few feature types are selected and extracted. Many of them have used STFT coefficients, which might not be sufficient to achieve accurate results. Evaluating different types of features, and selecting the most useful ones, is one of the advantages of this paper’s proposed activity detection framework.

The concept of data fusion has previously been used by researchers to integrate different types of data and overcome the limitations of merely using single data sources. Garcia-Ceja et al. [51] fused audio and sensor data using a multi-view stacking method to recognize different types of human activities. They suggested that the multi-view stacking method generates better results compared to aggregating features. They have used their method for human activity detection, which occurs in less noisy and complex environments as compared to construction job sites.

The presented research study in this paper is the first attempt towards using audio-kinematic sensor fusion to automatically detect multiple equipment activities with high accuracy. The contribution of this work to the existing body of knowledge is two-fold: first, by fusing audio with kinematic signals for multiple activity detection, and second, by using different types of time-domain and frequency-domain features.

## 3. Materials and Methods

The proposed hybrid system in this paper utilizes audio and kinematic data to detect multiple activities of construction equipment. Audio recordings from a microphone (placed outside the cabin) and kinematic recordings from IMUs (accelerometer and gyroscope embedded in a mobile phone placed inside the cabin) are used together to obtain accurate results. As explained in the previous section, each of the audio and kinematic signals has specific advantages and drawbacks. Microphones can cover large areas of job sites without needing to be directly attached to a specific piece of machinery. It is also well known that audio patterns generated by construction equipment are often independent of the operator and the orientations and directions followed by the machine [1]. On the other hand, IMU sensors shall be connected directly to the equipment and are capable of detecting almost every minor motion of the machine during operation. As a result, integrating these two data sources will help overcome the drawbacks of each one while taking advantage of the other source of data.

**Figure 1:**
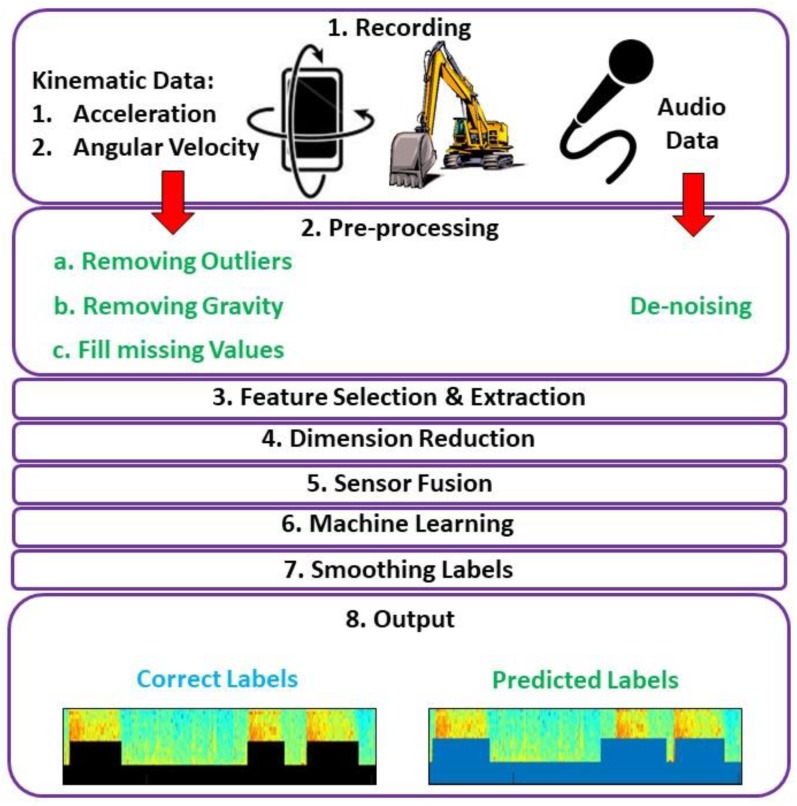
An overview of the hybrid kinematic-acoustic system for activity detection of construction machinery.

As indicated in Figure 1, the proposed hybrid system consists of the following major components: (1) recording both audio and kinematic signals, (2) pre-processing data using de-noising algorithms, (3) selecting and extracting different audio- and kinematic-based features, (4) filtering less-useful features or reducing the dimension of the extracted feature sets, (5) sensor fusion using both audio and kinematic data, (6) training and testing a Support Vector Machines (SVM) model using extracted features, and (7) smoothing labels. These steps are explained in more detail in subsequent sections.

### 3.1. Recording Audio and Kinematic Signals

**Figure 2:**
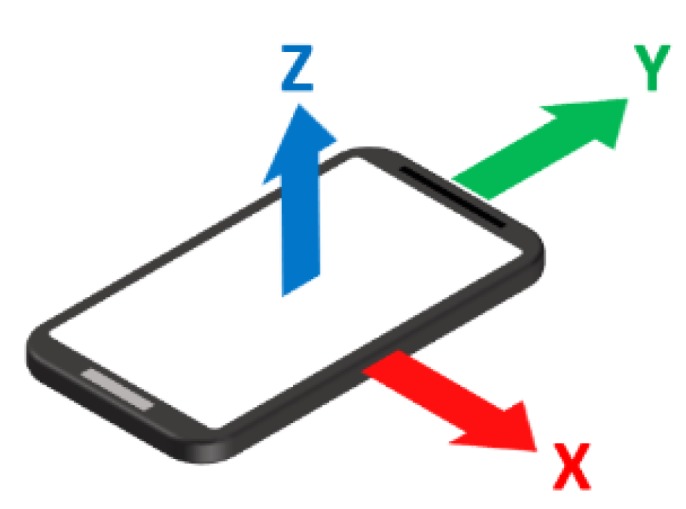
X, Y, and Z orientation axes relative to a typical mobile phone.

The first step toward implementing the presented activity detection system is collecting and recording input data. In this paper, the authors have recorded two types of data: (1) audio: This data is recorded using a microphone placed outside the cabin, and (2) kinematic: This data is recorded using IMUs embedded in a mobile phone inside the cabin. Technical details about the devices used in this paper are described in the experimental setup section. In this paper, two types of kinematic data are used: (1) acceleration data (Ax, Ay, and Az), and (2) angular velocity data (Vx, Vy, and Vz). The following illustration shows the X, Y, and Z orientation axes relative to a typical mobile phone (Figure 2). Kinematic data is recorded using accelerometer and gyroscope sensors embedded in the mobile device. Because the starting point of audio and kinematic data might be slightly different for time synchronization between audio and kinematic signals, an equipment horn has been used to generate a sound and vibration. This short high-pitched sound is easily recognizable throughout the signal and could be used as the starting point of recording for synchronization. Also, a video camera constantly records the entire scene, capturing all of the activities performed by the equipment over time. This video will be further used as the benchmark in order to label actual equipment activities.

### 3.2. Pre-Processing Data

The recorded data contains noisy and unintended signals (e.g., gravity for kinematic data) which could negatively affect the proposed activity detection system’s performance. These unwanted signals can be eliminated or diminished by utilizing some efficient algorithms. In this research, two different algorithms are used separately for the audio and kinematic signals.

To reduce unwanted background noise from the audio signal, the authors have implemented a signal enhancement algorithm developed by Rangachari and Loizou [52]. This algorithm has been used in similar studies [1] and it is proven to be an efficient method. Their method is capable of reducing noises in complex environments along with minimum distortion of the desired signals.

A similar process is also required for kinematic signals. The developed noise reduction process for kinematic signals consists of the following steps:

1. Removing gravity: This step is only applied on an accelerometer sensor because its values are subject to both dynamic (or external) and static (or gravity) accelerations. Thus, gravity components need to be eliminated from the signal. Equation (1) shows a low-pass filter for accelerometer sensor values. The cut-off frequency of 0.1 to 0.5 Hz is recommended to remove the gravity component from the data [53,54]. This equation calculates g-values for the sensor (g is initially set to zero) and then in Equation (2), g-values are subtracted from the sensor values. More details about these equations can be found in Bayat et al. [55].
  g(t) = (1 − a) × g(t − 1) + a × s(t) and g(0) = 0,(1)
  s(t) = s(t) − g(t),(2)
  where, g(t) is the gravity value, which is initially set to zero and is updated accordingly, the parameter s is accelerometer sensor amplitude, the parameter t is time, and the parameter a is a variable between 0 and 1 that controls the cutoff of the filter. In similar applications for removing gravity, the value of a is set to 0.1. By implementing these equations, the effect of gravity is removed and the actual kinematic values representing relative movements with respect to the equipment’s cabin can be derived.
2. Removing outliers: Outliers are unwanted noise or behaviors significantly different from the rest of the data. They decrease the accuracy of the system, so they should be eliminated. Data smoothing consists of techniques for removing these data points. Moving window methods are utilized to analyze data in smaller groups at a time. In this paper, the authors tested the moving window medians of length 3, 6, 12, and 24 using cross-validation. It is found that the window size of 3 is more effective to detect outliers.
3. Filling missing values: During recording data, and due to connection issues, some data points might be missed or not be recorded. These missing values affect the accuracy of the system. Thus, it is crucial to find a way to fill these values. The method for filling missing values is the same as detecting and removing outliers. Similar to the chosen window size for removing outliers, the authors tested different window sizes and found that window size 24 has less impact on the data.

**Figure 3:**
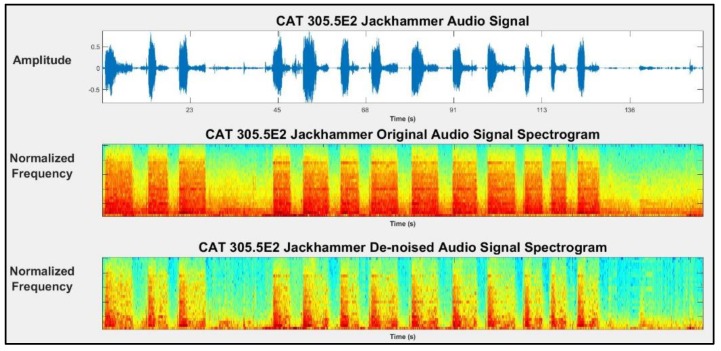
A sample of an audio signal and its respective spectrograms before and after de-noising (machine: jackhammer 305.5E2).

Figure 3 demonstrates sample plots for acceleration (Ax) and audio signals with their respective spectrograms before and after de-noising for a jackhammer 305.5E2. 

After both audio and kinematic signals are de-noised and refined, they need to be synchronized. In this paper, the authors have synchronized data manually. Using the generated sound and vibration from the equipment horn, a similar signal spike is detected on both audio and kinematic signals. Also, their respective spectrograms were identified and previous points were then cropped and shifted in a way that allowed both signals to have the same starting point. As a result, both signals were synchronized and could be further processed, as described in the following sections. 

### 3.3. Selecting and Extracting Features

**Figure 4:**
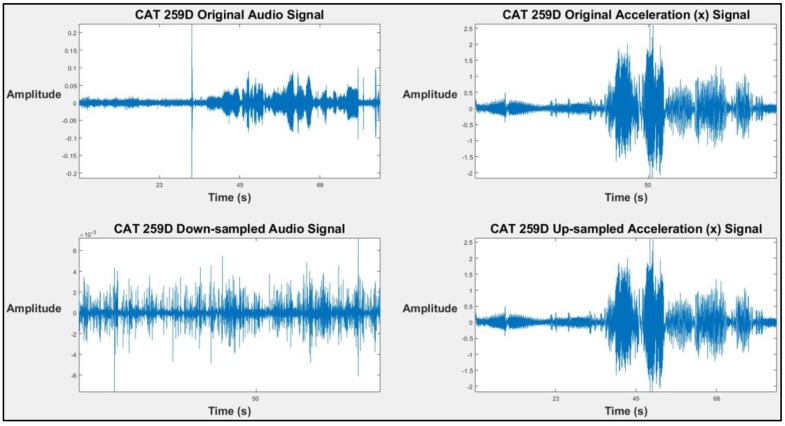
The original and resampled signals for both down-sampling and up-sampling options (machine: Caterpillar (CAT) 259D).

The same number of bins are required for feature fusion in the next steps to integrate both audio and kinematic signals. Because of different sampling frequencies of audio and kinematic signals (44,100 Hz for the audio signal and 100 Hz for the kinematic signal), the corresponding number of data points also differ. Thus, in order to obtain the same number of bins, two options are available: (1) the audio data can be down-sampled, or (2) the kinematic data can be up-sampled. These two options are evaluated and it is found that the up-sampled signal has the same amount of information [56], but down-sampling the audio data leads to information loss and signal distortion. All of the frequency components greater than half the new sampling rate ($\geq \frac{f_{s}New\;\left(=100\;\mathrm{Hz}\right)}{2}=50\;\mathrm{Hz})$ need to be removed using a low-pass filter before applying the down-sampling process to avoid aliasing. This process removes most of the useful frequencies in the audio signal and distorts the signal. In Figure 4, a comparison between the down-sampling and up-sampling effect is shown. The original signal and the resampled signal for both scenarios are shown. It is clear that the down-sampled audio signal is distorted. Also, the authors evaluated both methods and found that down-sampling the audio signal decreases the accuracy of the model up to 20% with respect to up-sampling the kinematic signals. Therefore, the linear data interpolation function called “interp1” in MATLAB is used to up-sample the kinematic signal, from 100 Hz to 44,100 Hz.

Interpolation is used to make the data points equivalent for both signals. The Linear 1-D data interpolation function called “interp1” in MATLAB is used to increase the number of data points for kinematic signal, from 100 data points per second to 44,100 data points per second. These new interpolated data points, which now both have a sampling frequency of 44,100, are used for further processes.

Now, both audio and kinematic signals have the same number of data points. For feature extraction, audio and kinematic signals were divided into short time segments and these segments were converted into a time-frequency domain representation using the STFT method. In this paper, STFT implementation in MATLAB using a Hanning window size of 512, with 50% overlap, was utilized to extract features, as described in previous papers [1,2,57]. This particular window size was chosen because it provides enough time resolution, as indicated by Cheng et al. [1]. Also, only the magnitudes of Short Time Fourier Transform (STFT) coefficients have been considered as features.

Another challenge during the feature extraction procedure is that some features work fine with one of the audio or kinematic data, while other features could work well with both. To take this point into account, different types of features in the time and frequency domains were considered for each of these signals, as shown in Table 1. These features were selected and tested for different types of equipment and job sites, and have been proven effective in class separability. STFT Coefficients have been used in Reference [1] and demonstrated satisfactory performance in the system. Root Mean Square (RMS) and Short Time Energy (STE) are related to signal energy, which had already shown accurate results in previous studies [58]. As described in Reference [59], the engine sound often has significant spectral components, which could help better identify different machine activities. Spectral Centroid (SC), Spectral Roll-Off (SRO), and Zero Crossing Rate (ZCR) were chosen in a previous research project for automatic vehicle and engine classification based on audio data [60]. Spectral Flux (SF) is another desired feature that has been used in similar research for vehicle engine classification [61]. All of these spectral features, such as Spectral Entropy (SE), were previously implemented to detect the presence of and identify specific types of vehicles in traffic using acoustic signals [62]. 

### 3.4. Dimension Reduction

**Figure 5:**
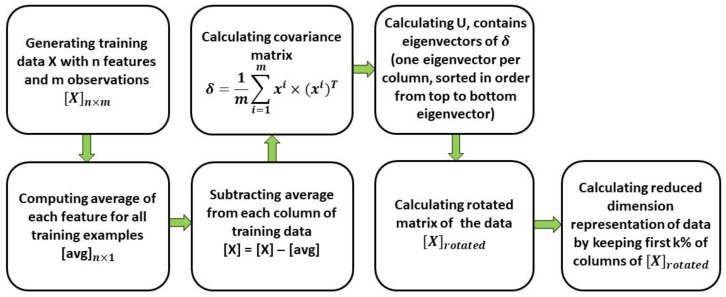
The proposed dimension reduction algorithm using Principal Component Analysis (PCA).

In the Machine Learning area, dimension reduction is the process of reducing the number of features extracted from the training data. This process is particularly important for a robust activity detection and monitoring system, as the results should be eventually generated in real-time (or near real-time setting). To achieve this goal, and as demonstrated in Figure 5, the Principal Component Analysis (PCA) algorithm has been implemented to reduce the dimension of the training data. PCA is an efficient method commonly used to highlight variations and bring out strong patterns in a dataset, by transforming the feature space using centering and rotation such that the resulting vectors are pointing in the direction with the highest variance in descending order. Walse et al. [63] utilized PCA before feeding the features into a DNN model for human activity recognition using mobile sensors data. They have reported that PCA can decrease the computational time significantly, which can further be used for real-time purposes. Before using PCA, the extracted features are normalized using z-score to avoid scaling effects, while ensuring that any feature with larger domain will not dominate features with smaller domain [64,65,66]. A feature value X of a feature F is normalized to $X^{\prime }$ using Equation (3):

(3) $$X^{\prime }=\frac{X-\mu \left(F\right)}{\sigma \left(F\right)}$$

where, X is the feature value, μ(F) is the arithmetic mean of all values of feature F, σ(F) is the standard deviation of all values of feature F, and $X^{\prime }$ is the normalized feature value.

**Figure 6:**
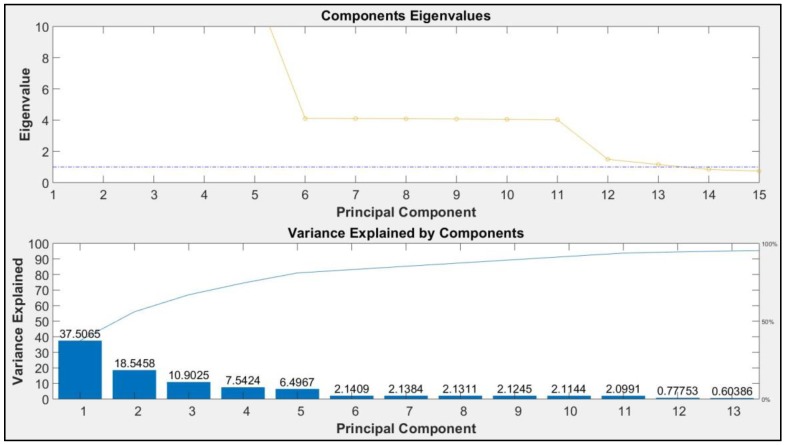
Most discriminative principal components and the variance they explain.

PCA aims at finding the most discriminate principal components in the orthonormal eigenvector space. The eigenvalues of the covariance matrix of the data provide the variance at each orthogonal direction (eigenvectors). In this paper, 32 features from each kinematic data type (totally 6×32) and 31 features from audio data have been extracted. As a rule of thumb, the Kaiser–Guttman rule [67] states that the principal components with eigenvalues greater than 1 should be retained. In this paper, the authors used this rule, and as a result, we found that 13 principal components have eigenvalues greater than 1, which cumulatively explain 95.12% of the variance. In Figure 6, the top plot shows the principal components and their eigenvalues. The eigenvalues for the first 13 components are greater than 1. Also, in the bottom plot, the principal components and the explained variance for each of them is shown. These components are used for the next steps.

### 3.5. Sensor Fusion

**Figure 7:**
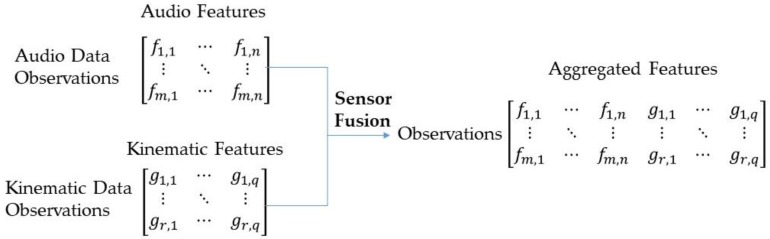
Sensor fusion using the feature aggregation method.

In order to evaluate the performance of the proposed hybrid system, three scenarios were taken into account: (1) using audio signals only, (2) using kinematic signals only, and (3) integrating audio and kinematic signals. For the first and second scenarios, only audio and kinematic features were extracted respectively, and fed into the model. For the third scenario, a feature fusion procedure was implemented to integrate both audio and kinematic signals for training and testing the SVM model. In other words, extracted feature matrices from audio and kinematic data are combined to obtain a single feature matrix, which is more discriminative than any of the input feature vectors. It is a common practice to fuse multiple sources such as audio, video, and text to improve the accuracy of the system and generate useful information. The most common method for sensor fusion in the domain of wearable sensors is aggregation, which means that instead of training classification models for each data type, different feature sets from different sources are concatenated to obtain a single feature set for training a single classification model. Figure 7 demonstrates the process of sensor fusion using the feature aggregation method. In Figure 7, columns show the feature sets and rows representing the observations (data points). Parameters “m” and “g” show the number of data points for audio and kinematic data, which are equal using the method explained in Section 3.3. As explained in the next section, this feature set was further used to train and test the SVM model. A detailed explanation of this fusion process can be found in Reference [51].

### 3.6. Support Vector Machines (SVM) Model

**Figure 8:**
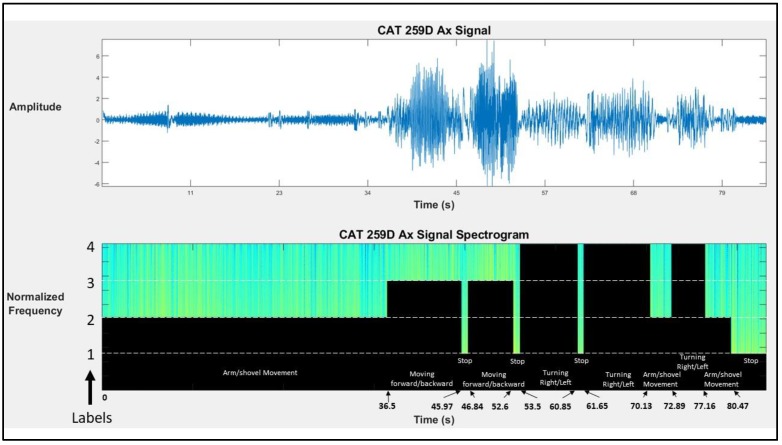
CAT 259D actual labels.

An SVM model with Radial Basis Function (RBF) kernel was utilized for predicting activities of construction machines. This machine learning classifier has been tested in previous studies conducted by the authors [1,2,57] and was found efficient and accurate. For comparison purposes and by utilizing the video recordings as a benchmark, all different periods of audio and kinematic signals were identified and labeled accordingly. Table 2 demonstrates a chronological list of activities performed by a CAT 259D compact track loader. A visual representation of the corresponding activities for this specific case study is also depicted in Figure 8. The black bars on the bottom plot of Figure 8 are the actual labels for activities 1 to 4. It is necessary to mention that the authors used 90% of available data for training and the remaining 10% for testing the developed machine learning classifier. Generated labels in this step are fed into the next step for post-processing.

### 3.7. Smoothing Labels

**Figure 9:**
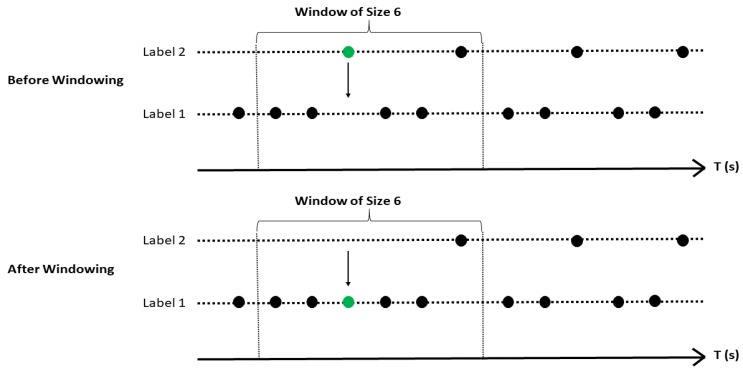
A sample window filtering process.

Because the labels generated by SVM are fluctuating, they cannot precisely represent their related activities. So, in order to visualize the predicted labels accurately, three smoothing methods were applied: (1) Small Window Filtering (SWF), (2) Big Window Filtering (BWF), and (3) Markov Chain Filtering (MCF). The *y*-axis shows the labels corresponding to their related activities and the *x*-axis shows the time bins. The first two algorithms use Moving Average Window (MAW) and the only difference between them is the size of the window utilized by each of them. The window sizes for SWF and BWF are determined as 2 and 6 with a threshold of 0.68. These window sizes and threshold are chosen based on 10-fold cross-validation, which leads to the most accurate results. As a simple example, the process of window filtering is shown in Figure 9. In this figure, the window size of 6 is shown. The algorithm calculates the ratio in Equation (4), which is $\frac{5}{6}=0.83$, and changes its label from “Label 1” to “Label 2”, because it has a ratio higher than 0.68.

(4) $$\mathrm{Smoothed}\;\mathrm{Label}=\{\begin{array}{ll} Label\;2,\; & if\;ratio=\frac{Count\;of\;Label\;2\;in\;the\;Window}{Total\;Count\;of\;Labels\;in\;the\;Window}\geq 0.68\\ Label\;1,\; & otherwise \end{array}$$

**Figure 10:**
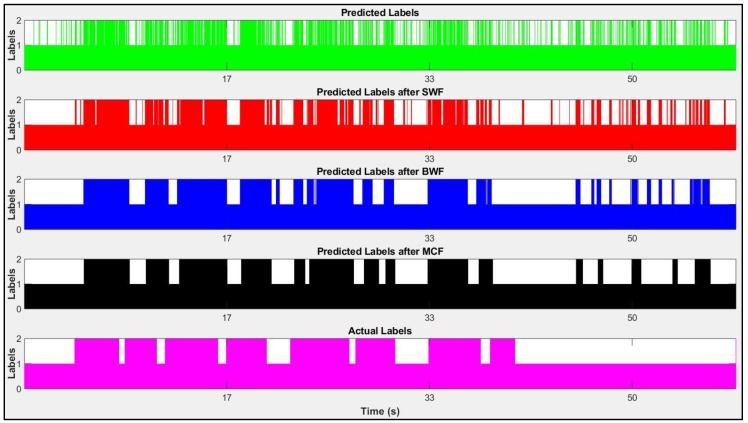
From top to bottom: (1) predicted labels (**green**), (2) predicted labels after Small Window Filtering (SWF) (**red**), (3) predicted labels after Big Window Filtering (BWF) (**blue**), (4) predicted labels after Markov Chain Filtering (MCF) (**black**), (5) actual labels (**magenta**). Black labels represent the activities more realistic than the blue, red, and green ones.

The third method uses the MCF to predict the next label, based on the previous label state. MCF scans through the labels, predicted from the BWF again, to make the labels smoother. The transition probability matrix for MCF is determined based on ground truth data, which specifies how the state (activity label) evolves over time. The reason that MCF labels are more realistic (i.e., they contain less noise and conform better to the actual labels) is that the MCF uses the ratio that BWF has calculated in the previous step (i.e., 0.83) as its threshold, not the threshold defined first (i.e., 0.68), which is higher and makes the MCF less sensitive to local changes. A comprehensive elaboration of these methods is presented in a paper authored by Sabillon et al. [15]. In this paper, the authors have used the smoothed labels of MCF, because the output results of SWF and BWF were not accurate enough (Figure 10).

## 4. Experimental Setup and Results

**Figure 11:**
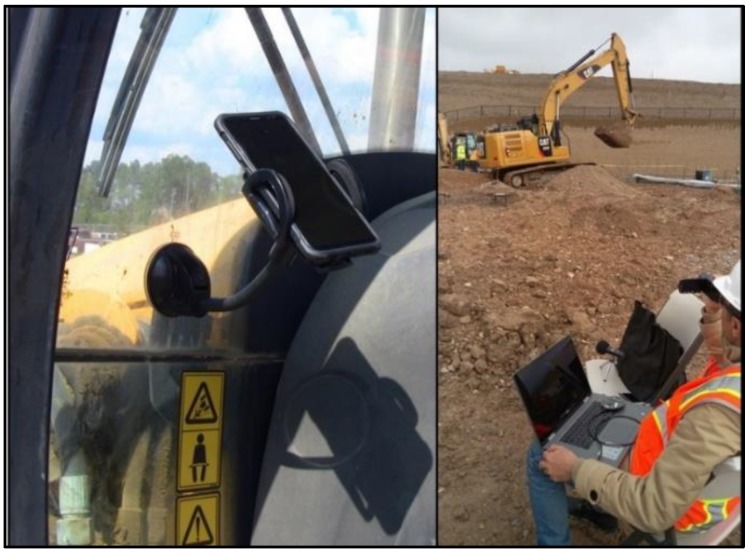
Configuration of audio and kinematic recording devices in a sample job site.

The aforementioned hybrid system has been evaluated under various conditions, such as different types of equipment and job sites. In this paper, the authors collected and processed data of 10 pieces of construction machines working at three different job sites: (1) jackhammer 305.5E2 (stop, drilling, rotating/moving arm, and moving forward/backward), (2) CAT 259D (stop, arm/shovel movement, moving forward/backward, and turning right/left), (3) SkyJack SJ6826 (maneuvering forward/backward, and raising/lowering), (4) XTREME 842Lift (stop, moving forward/backward, and moving arm), (5) CAT 308E (scraping and moving/rotating arm), (6) CAT 305.5E2 (extending arm and rotating cabin), (7) Dozer 850K (stop, moving forward, and moving backward), (8) Concrete Truck (pouring concrete and moving forward/backward), (9) CAT 938M (stop, moving forward/pushing soil, and moving backward), and (10) CAT 210G Vibrator (stop and vibrating). All of the activities of these machines during certain periods were observed, and the audio and kinematic signals generated were recorded accordingly. The collected audio and kinematic signals were stored as vectors and then exported to a MATLAB platform for further analysis. A Zoom H1 digital handy recorder and an iPhone 6s Plus have been used for data collection purposes. The selected off-the-shelf microphone was located within 50 feet from the desired equipment on a fixed tripod to avoid any movement noises. To record kinematic signals, the iPhone had been placed inside the operator’s cabin and mounted to fix its position and prevent any unwanted noise signals from the phone. Figure 11 shows the configuration of the devices in a sample job site. Furthermore, a video camera was utilized for recording all activities of equipment and producing ground truth data. Finally, an equipment horn was used to generate a short distinct sound to properly set the recording beginning point. 

The recorded audio and kinematic signals are pre-processed. Then, the aforementioned features are extracted and using PCA, the dimension of the feature set is reduced. In the next step, these features are fused using the feature aggregation method. Next, the aggregated features are fed into the SVM model to determine different activities. Finally, the accuracy of the proposed system has been evaluated by comparing detected labels with actual labels.

**Figure 12:**
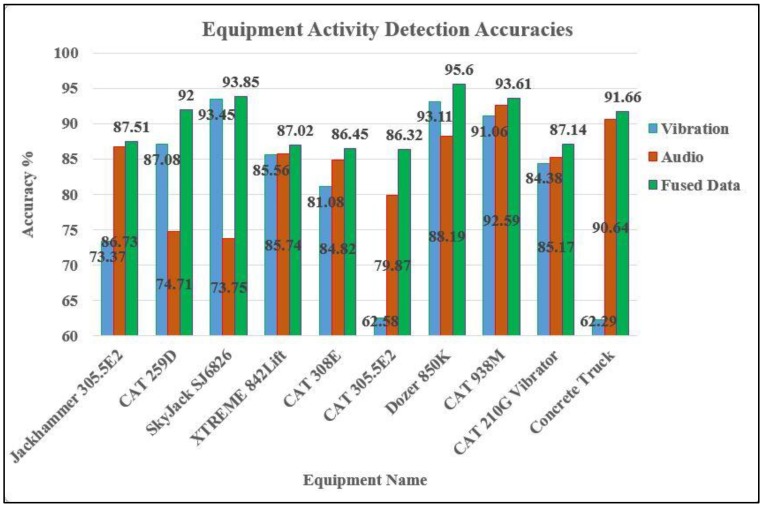
A summary of accuracy rates for activity detection of 10 different pieces of construction equipment.

To quantify the accuracy of labeling, confusion matrices were completed for each piece of equipment. These matrices illustrate how the predicted labels are precise when compared with their correlated actual labels. Accuracy, defined as the ratio of correctly predicted labels to the total number of labels, has been separately calculated for kinematic signals, audio signals, and fused data in this paper. Table 3 presents the results of calculating confusion matrices for a CAT 259D, with different accuracies for activity detection using kinematic signal, audio signal, and fused data. The value for the kinematic signal is the accuracy level after fusing all six components of acceleration and angular velocity sensors, which are then fused with the audio signal. Similarly, confusion matrices for other types of equipment have been calculated, and their related accuracies are presented in Figure 12.

## 5. Discussion

### 5.1. Audio or Kinematic Data?

Careful analysis of the results (presented in Figure 12 and Table 4) verifies the hypothesis that every single type of kinematic or acoustic data would generate more accurate results under certain scenarios. For example, the concrete truck mixer generates very identifiable sound patterns while pouring concrete, so audio is an excellent source of data for this category of equipment’s activity classification. At the same time, a kinematic sensor placed inside the cabin is not able to collect reliable data due to the distance and lack of direct connection between the cabin and the discharge chute. Therefore, distance between the kinematic sensor and the signal source is a major factor, which needs to be considered when working with kinematic sensors. Alternatively, for certain types of equipment such as the skyjack SJ6826, kinematic signals are a great source of input data for activity detection purposes because moving forward/backward and raising/lowering the arm does not generate distinct sound, and only their corresponding kinematic signals are detectable. For jackhammer 305.5E2, both data sources are appropriate for activity detection because drilling rocks generates sufficient vibration and sound signals. A similar situation exists for Dozer 850K, where either kinematic or acoustic signals could provide accurate results. The case of the CAT 305.5E2 was challenging, as neither audio nor kinematic data could individually produce accurate results. 

To sum up, excavators (e.g., CAT 308E and CAT 305.5E2) generate sufficient sound, which makes audio a better source of input data for activity detection purposes. On the other hand, for bulldozers (e.g., Dozer 850K) kinematic data is a better option, similar for lifts (e.g., skyjack SJ6826 and XTREME 842). For skid-steer loaders (e.g., CAT 259D), both data sources are capable of generating reliable results (Table 4). In Table 4, low, moderate, and high columns show accuracies of 0–75%, 75–85%, and 85–100%, respectively.

### 5.2. Role of Data Fusion

As indicated in Table 4 and Figure 12, fusing both audio and vibration data provides a significant improvement in results. Considering the results of implementing the proposed system, it is possible to achieve up to 20% higher accuracy rates by fusing acoustic and kinematic data.

Other than the issues discussed in the previous section, “time synchronization” is an essential cause for obtaining less accurate results. The recorded audio and kinematic signals do not have the same starting points and durations. The synchronization issue has been addressed through a manual procedure, as explained in Section 3.2. The authors intend to develop an automated synchronization procedure as part of their future research plans.

### 5.3. Applications of Equipment Activity 

Recognizing activities of construction equipment might not be useful. In other words, it is the basis for performance monitoring of construction job sites. Construction managers are interested in recognizing equipment activities due to its potential applications for construction performance monitoring. The potential outcomes of activity recognition are as follows:

1. Maintenance Assessment: Collecting data from different sensors and recognizing equipment activities can further be used as a platform for monitoring its abnormality or well-being, fuel consumption evaluation, and utilization time and cycle time estimation. Using an automated real-time framework, construction managers are able to continuously monitor activities of equipment using a standalone device or a mobile app. They can be provided with productivity rates and make proper decisions based on the performance of the equipment. For example, they can be notified if an equipment productivity rate is low and make proper decisions such as repairing the equipment, changing the equipment, or even changing the equipment operator.
2. Environmental Performance Monitoring: Construction equipment usually releases detrimental smoke, which makes the construction job site unhealthy and unsafe for personnel. Also, it has harmful impacts on the environment. Thus, continuous monitoring of equipment can help construction managers track the emissions of the equipment during its operations and find its potential deficiencies. In other words, engine audio and kinematic data of equipment can be compared with the new models’ data and identify any abnormality to prevent more pollution.

## 6. Conclusions

In this paper, the authors propose a robust hybrid equipment activity detection system based upon using both audio and kinematic data. The proposed method is validated using ten types of equipment and the obtained results were promising. The authors demonstrated the capabilities of a hybrid system in overcoming limitations of single sources of data and generating more accurate results for a broader range of construction equipment. Several efficient pre-processing algorithms (i.e., de-noising, removing gravity, removing outliers, and filling missing values) were implemented in this paper to refine the data and improve the overall performance of the proposed system. Considering the computational efficiency of the implemented algorithms, the proposed method is also capable of being used in near real-time scenarios and construction operations can be monitored continuously without the need for human interference. In Table 5, computational time for testing three types of equipment for different types of data is shown. As illustrated here, the computational time is not directly related to the number of activities and is less than one second, which could provide an indication of possible use for near real-time applications.

Furthermore, activity recognition of construction equipment is the first step toward construction operation monitoring and it can help construction managers in different aspects such as productivity analysis, equipment downtime/idle time detection, equipment cycle time estimation, and equipment fuel use control.

Following are some of the key contributions of this research:

1. Construction job sites may vary in types of existing equipment, weather, complexity, etc. Some types of equipment, especially new models, might not generate kinematic signals and this would make it almost impossible to detect activities. Also, distance and inaccessibility of equipment or the presence of sound barriers may hinder the process of recording audios. Rainy, big and crowded job sites might affect the accuracy of each data type and decrease the precision of the detection. In this paper, both audio and kinematic signals have been fused to overcome and cover these limitations.
2. Most of the aforementioned papers utilized a few features to train the machine learning model. In this paper, different types of time-domain and frequency-domain features were selected and evaluated before using in a training model. Also, a dimension reduction method has been implemented on the feature set to decrease the correlation between features and increase the class separability of features’ values. Moreover, it decreases computational time of the process which can further be used in real-time.
3. Different types of pre-processing algorithms were implemented in this paper on audio and kinematic signals which refine the data before being used in subsequent steps.

The focus of this research has been on activity analysis of single machines. The more realistic yet challenging scenario occurs when multiple machines operate simultaneously on a job site. For multiple machine recordings, sound and vibration signals from different machines come from different directions. A robust source detection and activity recognition algorithm must respond to sound and kinematic signals from a specific direction and block most of the noise outside the direction of interest. Addressing this important issue would be this research project’s future extension. In addition, and as part of future studies, the authors will investigate the use of robust PCA, because it has less sensitivity to outliers and it might increase the accuracy of feature reduction.

## Figures and Tables

**Table 1:** Extracted features.

Audio and Kinematic	Only Kinematic
25 Short Time Fourier Transform (STFT) Coefficients	Zero Crossing Rate (ZCR)
Root Mean Square (RMS)	
Short Time Energy (STE)	
Spectral Flux (SF)	
Spectral Entropy (SE)	
Spectral Centroid (SC)	
Spectral Roll-Off (SRO)	

**Table 2:** CAT 259D activities and the corresponding list of actual labels.

Activity	Start (s)	End (s)	Duration (s)	Activity Label
Arm Raising	0	13.4	13.4	2
Arm Lowering	13.4	24.4	11	2
Shovel Lowering	24.4	30.4	6	2
Shovel Raising	30.4	35.4	5	2
Arm Lowering	35.4	36.5	1.1	2
Moving Forward	36.5	45.97	9.47	3
Minor Stop	45.97	46.84	0.87	1
Moving Backward	46.84	52.6	5.76	3
Minor Stop	52.6	53.5	0.9	1
Turning Right	53.5	60.85	7.35	4
Minor Stop	60.85	61.65	0.8	1
Turning Left	61.65	70.13	8.48	4
Arm Raising	70.13	72.89	2.76	2
Turning Right	72.89	77.16	4.27	4
Arm Lowering	77.16	78.84	1.68	2
Shovel Lowering	78.84	80.47	1.63	2
Stop	80.47	87	6.53	1
End	87			

**Table 3:** Confusion matrix for CAT 259D.

			Actual Label				Accuracy %
			Stop	Arm/shovel Movement	Moving Forward/Backward	Turning Right/Left	
Predicted Label	Vibration	Stop	41	58	21	2	87.08
		Arm/shovel movement	19	692	6	16	
		Moving forward/backward	19	15	227	1	
		Turning right/left	9	22	1	314	
	Audio	Stop	11	94	10	7	74.71
		Arm/shovel movement	3	690	19	21	
		Moving forward/backward	5	34	164	59	
		Turning right/left	1	73	44	228	
	Fused Data	Stop	66	41	10	5	92.00
		Arm/shovel movement	17	697	1	18	
		Moving forward/backward	6	4	252	0	
		Turning right/left	2	11	2	331	

**Table 4:** Classification accuracy.

	Vibration Accuracy			Audio Accuracy			Fused Data Accuracy		
	Low	Moderate	High	Low	Moderate	High	Low	Moderate	High
Jackhammer 305.5E2	✔					✔			✔
CAT 259D			✔	✔					✔
Skyjack SJ6826			✔	✔					✔
XTREME 842Lift			✔			✔			✔
CAT 308E		✔			✔				✔
CAT 305.5E2	✔				✔				✔
Dozer 850K			✔			✔			✔
CAT 938M			✔			✔			✔
CAT 210G Vibrator		✔				✔			✔
Concrete Truck	✔					✔			✔

**Table 5:** The computational time of different data types for real-time purposes.

		Testing Time (s)						
			Predicting Time			Total Time		
(1) Equipment	(2) Number of Activities	(3) Capturing and Pre-processing Data	(4) Vibration Data	(5) Audio Data	(6) Fused Data	(3) + (4) Vibration Data	(3) + (5) Audio Data	(3) + (6) Fused Data
Concrete Truck	2	0.918	0.043	0.008	0.046	0.961	0.926	0.964
XTREME 842Lift	3	0.963	0.030	0.020	0.015	0.993	0.983	0.978
CAT 259D	4	0.664	0.191	0.035	0.083	0.855	0.699	0.747
